# Impact of Physician Patient Load on Imaging Use in the Emergency Department

**DOI:** 10.5811/westjem.63096

**Published:** 2026-09-24

**Authors:** Wayne A. Martini, Jessica Monas, Nicole R. Hodgson

**Affiliations:** Mayo Clinic Arizona, Department of Emergency Medicine, Phoenix, Arizona

## Abstract

**Introduction:**

Emergency department (ED) crowding may alter physician decision-making, yet little is known about how real-time physician workload affects diagnostic imaging use. We examined the association between physician patient load and the proportion of patients who received diagnostic imaging, including computed tomography (CT), CT with intravenous (IV) contrast, CT without contrast, plain radiography, magnetic resonance imaging (MRI), and ultrasound (US).

**Methods:**

We conducted a retrospective cohort study of all ED visits at an academic tertiary care center (January 2019–December 2024). Physician workload was defined as the sequential patient assignment order per physician per day (range 1–17). Our primary outcome measures were binary indicators of whether each imaging modality was ordered during the ED visit: CT (any); CT with IV contrast; CT without IV contrast; radiograph, MRI; and US. Multivariable logistic regression with physician-clustered robust standard errors estimated adjusted odds ratios (aOR) for each imaging modality, controlling for age, sex, triage acuity, chief complaint, and temporal factors. We also performed mixed-effects logistic regression with physician random intercepts and a within-between (Mundlak) decomposition. Absolute risk differences (ARD) were computed for the 17th versus the first patient.

**Results:**

Among 287,925 encounters across approximately 41,132 physician shifts managed by 77 physicians, higher workload was associated with decreased odds of imaging for most modalities. Computed tomography with IV contrast showed the strongest effect (aOR 0.990, 95% CI, 0.986–0.994; within-physician aOR 0.991; ARD −2.2 percentage points [pp], from 21.8% to 19.6%). Results were as follows: any CT, aOR 0.994 (0.991–0.997); ARD −1.9 pp (from 38.7% to 36.8%); radiograph, aOR 0.994 (0.990–0.998, *P* < .001); ARD −1.6 pp (from 44.2% to 42.6%); US, aOR 0.994 (0.990–0.998, *P* < .001); ARD −0.7 pp (from 9.9% to 9.2%); and MRI was nonsignificant in the pooled model (aOR 0.993, *P* = .09) but significant in the mixed-effects model (*P* < .001). Noncontrast CT was unaffected (aOR 1.000, *P* = .87). Intraclass correlation coefficients ranged from 0.020–0.047, indicating 2–5% of imaging variance was attributable to physician-level differences.

**Conclusion:**

Higher physician workload was associated with modestly reduced imaging use, particularly for resource-intensive modalities. Noncontrast CT was unaffected. Within-physician analyses were consistent with the interpretation that these effects reflect real-time behavioral adaptation rather than physician practice style alone. These findings highlight a trade-off between throughput and diagnostic testing that warrants further study of its impact on clinical outcomes.

## INTRODUCTION

Diagnostic imaging use in the emergency department (ED) has increased markedly over recent decades, raising concerns about overuse, radiation exposure, and throughput delays.^1^,^2^ Advanced imaging modalities such as computed tomography (CT) and magnetic resonance imaging (MRI) provide critical diagnostic information but are resource-intensive, expose patients to radiation or contrast agents, and frequently prolong ED length of stay.^3^ Prior studies have documented wide variation in imaging ordering rates among emergency physicians, with higher use not necessarily associated with better patient outcomes.^4^

Emergency department crowding and physician workload have become ubiquitous challenges in emergency medicine. As patient volume and acuity rise, emergency physicians may alter their clinical behavior in response to time pressures. One hypothesis is that during busy periods, physicians might increase imaging as a diagnostic shortcut, ordering tests “just to be safe” to compensate for reduced time at the bedside. Conversely, overwhelmed physicians might avoid or defer certain imaging to expedite care, effectively rationing time-consuming diagnostics.^5^,^6^ Understanding which pattern predominates is important for patient safety: If workload pressure leads to omission of necessary imaging, critical diagnoses may be delayed; if it reduces low-value testing, it may represent adaptive efficiency.

Despite considerable interest in ED crowding and resource use, there is limited direct evidence on the relationship between physician-specific workload and imaging use. Prior research has examined system-level crowding effects on length of stay, prescribing, and admission rates, and several large studies, predominantly conducted by radiology investigators using department-wide volume metrics, have reported that imaging orders rise with ED crowding or higher EP volume.^10^–^12^ By contrast, few studies have examined how a physician’s active patient load, in real time, influences their tendency to order diagnostic imaging on a per-encounter basis.^7^ We aimed to address this gap by examining a large sample of ED visits over a six-year period, leveraging detailed operational data to link each patient with their treating physician’s contemporaneous workload.

We hypothesized that increasing physician workload would be associated with reduced diagnostic imaging use overall, with the largest reductions occurring for resource-intensive modalities (CT with intravenous [IV] contrast, ultrasound [US], and MRI) and minimal change for time-critical modalities used for cannot-miss diagnoses (noncontrast CT for stroke or trauma, and plain radiography. We examined the association between physician patient load and the proportion of patients who received CT, CT with IV contrast, CT without contrast, radiograph, MRI, and US. Understanding these dynamics may help clinicians and administrators recognize potential decision-making shifts under workload pressure and guide strategies to optimize imaging use without compromising patient care.

**Population Health Research Capsule**

What do we already know about this issue?

*Emergency department (ED) imaging use varies widely, but real-time effects of physician workload on imaging decisions have not been well characterized*.

What was the research question?

*Does higher real-time physician workload change imaging use in the ED?*

What was the major finding of the study?

*The adjusted odds ratio (aOR) for computed tomography with contrast was statistically lower for the physician’s 17th vs. 1st patient of the shift (aOR 0.99 per +1 patient [95% CI, 0.986–0.994]; absolute risk reduction −2.2 percentage points)*.

How does this improve population health?

*Recognizing that crowding subtly reduces ED imaging may inform staffing and protocols that protect diagnostic care during high-load shifts*.

## METHODS

### Study Design and Setting

We performed a retrospective cohort study of all patient visits to an academic tertiary care ED (approximately 45,000–60,000 annual visits) over a six-year period from January 1, 2019, through December 31, 2024. The study site is in an urban metropolitan area; the institution does not hold a formal trauma center designation and hosts an emergency medicine residency program accredited by the Accreditation Council for Graduate Medical Education. Board-certified or board-eligible emergency physicians directly evaluate each patient and are responsible for all imaging orders; while residents and advanced practice clinicians may enter orders within the electronic health record (EHR), all imaging orders are placed under direct attending supervision and are attributed to the attending physician of record. Fewer than 10% of all cases during the study period were co-managed with a resident or advanced practice clinician. Patients are automatically assigned upon arrival to emergency physicians on a rotational basis, in practice removing physician discretion on when and which patients to accept. The study was reviewed and approved by the institutional review board with a waiver of informed consent due to its retrospective nature and minimal risk.

Our retrospective data collection methods adhered to recommendations for medical record review studies in emergency medicine as described by Worster et al.^8^ Specifically, we used standardized EHR data extraction with predefined variable definitions, minimizing abstractor subjectivity.

### Study Population and Data Sources

We extracted all ED patient encounters in the specified date range from the EHR. For each visit, we obtained patient demographics (age, sex), triage acuity (Emergency Severity Index [ESI] 1–5), chief complaint, arrival timestamp, the identity of the attending emergency physician, and indicators of imaging orders placed during the ED visit.

For each encounter, we determined the “physician assignment order,” defined as the sequential position of the encounter within the attending physician’s set of patients seen that calendar day. This served as a proxy for the physician’s real-time workload position within their shift; it captures how deep into a workload sequence the physician is when a given patient arrives but does not directly measure acuity-weighted simultaneous patient load (eg, the number of concurrently active patients) or nonclinical workload (eg, interruptions, calls, documentation backlog). Within each physician-day group, encounters were ordered chronologically. One modification accounted for overnight shifts: patients arriving at or after 10:30 pm were attributed to the next calendar day’s workload sequence.

Encounters with assignment order greater than 17 were excluded (*n* = 723, 0.2% of all visits). This ceiling reflects an institutional operational artifact rather than a clinical threshold: the automated rotational assignment system caps assignments at 17 per attending physician per shift, after which the system rolls to the next available physician or triggers a shift-handoff prompt. The excluded encounters therefore represent rare overflow situations driven by system handoff timing rather than by physicians being asked to see additional patients beyond their cap. An additional 4,695 (1.6%) encounters lacking a linked attending physician identifier were also excluded. The final cohort comprised 287,925 visits managed by 77 attending physicians.

### Variables and Definitions

The primary independent variable was physician assignment order (1–17), treated as a continuous predictor reflecting the incremental effect of being one position later in the physician’s queue. Outcomes were binary indicators of whether each imaging modality was ordered during the ED visit: CT (any); CT with IV contrast; CT without IV contrast; radiograph; MRI; and US (including both point-of-care and formal radiology US). These were not mutually exclusive; a single visit could involve multiple modalities. Covariates included patient age (continuous), sex (male, female, other/unknown), ESI level (categorical, with ESI 3 as reference), chief complaint (top 10 presenting complaints, plus “other” as reference), arrival hour (0–23, continuous), a binary night shift indicator (arrival time ≥ 10:30 pm), and a weekend indicator.

### Statistical Analysis

We first performed descriptive analyses to characterize the cohort and the unadjusted relationship between physician assignment order and imaging use. We tabulated imaging frequency by modality and examined imaging rates stratified by workload groupings, using chi-square tests for trend. Our primary analysis used pooled multivariable logistic regression with Huber-White robust standard errors clustered by physician. Six separate models were fit, one for each imaging modality, producing an adjusted odds ratio (aOR) for the effect of a one-patient increase in assignment order on imaging odds, controlling for the covariates listed above.

We additionally employed two approaches to characterize physician-level heterogeneity. First, mixed-effects logistic regression (generalized linear mixed model [GLMM]) with a random intercept for each physician, fit using variational Bayes approximation (BinomialBayesMixedGLM in Python statsmodels). This partitions variance into within-physician and between-physician components and yields the intraclass correlation coefficient (ICC = σ^2^_u / [σ^2^_u + π^2^/3]), representing the proportion of total variance in imaging decisions attributable to the physician. Second, a within-between decomposition (Mundlak model) splitting the workload variable into a within-physician component (encounter-level deviation from that physician’s mean assignment order) and a between-physician component (the physician’s overall mean). This method cleanly separates within-physician behavioral adaptation under varying workload from between-physician practice style differences. A Hausman-type test assessed whether within and between coefficients differed significantly.

Absolute risk differences (ARD) were computed by comparing marginal predicted probabilities at assignment order 1 versus 17. Model diagnostics included variance inflation factors (all < 2.0) and c-statistics (area under the receiver operating characteristic curve; range 0.654–0.766). All hypothesis tests were two-tailed with α = .05. Analyses were conducted using Python, version 3.13, with pandas, statsmodels, and scikit-learn.

## RESULTS

### Characteristics of Study Subjects

From 2019 through 2024, there were 293,343 patient visits to the study ED. After excluding 723 encounters beyond the 17th assignment order and 4,695 (1.6%) encounters lacking a linked attending physician identifier, 287,925 ED visits remained in the study cohort, managed by 77 unique attending emergency physicians across approximately 41,132 physician shifts over the six-year period.

The median patient age was 61 years (interquartile range [IQR] 42–74; mean (standard deviation), 57.8 (20.7) years. A substantial proportion (44.0%) were elderly (≥ 65 years), and 2.1% were pediatric (< 18 years). Sex distribution was 53.3% female and 46.7% male. By triage acuity, ESI 3 was most common (59.2%), followed by ESI 2 (26.7%), ESI 4 (12.7%), ESI 1 (0.7%), and ESI 5 (0.6%). The median assignment order was 7; approximately 40% of encounters were order 1–5, 30% were ≥ 10, and 5% were ≥ 15. At the physician level, the median number of encounters per physician was 2,436 (range 1–12,752).

### Imaging Use

In unadjusted analyses, CT and US showed mild downward trends with increasing workload (chi-square trend *P* < .01), whereas radiograph and MRI rates were relatively flat across the workload spectrum (*P* > .10). Figure 1 illustrates the unadjusted imaging rates by assignment order grouping. Supplementary Figure S1 provides a complementary view of the same data plotted at the level of exact assignment order (1–17) with 95% confidence bands, allowing the per-patient dose-response to be examined directly.

### Adjusted Association Between Workload and Imaging

Table 1 and Figure 2 present the results of the multivariable analyses. After controlling for patient age, sex, triage acuity, chief complaint, and temporal factors, higher physician workload was significantly associated with reduced odds of ordering most imaging modalities. Adjusted odds ratios for all model covariates are reported in Supplementary Table S1. Briefly, higher triage acuity was the strongest single predictor of CT and MRI use (ESI 1 had several-fold higher odds than ESI 3), whereas ultrasound was ordered less often at the highest acuity; older age increased the odds of CT and plain radiography but not ultrasound; chest pain, abdominal pain, and fall-related chief complaints drove the largest complaint-specific imaging effects; and arrival hour and night-shift indicators contributed small, mostly nonsignificant effects. The workload effect estimates reported below are adjusted for all these covariates.

Computed tomography with IV contrast showed the strongest effect: aOR 0.990 per additional patient (95% CI, 0.986–0.994, *P* < .001), meaning approximately a 1.0% decrease in odds per patient. The within-physician effect from the Mundlak decomposition was 0.991, suggesting this reflects behavior change within individual physicians rather than solely between-physician differences. The between-physician effect was notably larger (aOR 0.933), indicating that physicians who habitually see more patients also tend to order fewer contrast CTs overall. The GLMM yielded the highest ICC among all modalities (0.047). Cumulatively, the 17th patient had 14.1% lower odds of contrast CT than the first patient (ARD −2.2 percentage points [pp], predicted rate 21.8% versus 19.6%).

Any CT imaging showed a similar pattern: aOR 0.994 (95% CI, 0.991–0.997, *P* < .001), within-physician aOR 0.994, ICC 0.044, and ARD −1.9 pp (38.7% to 36.8%). Plain radiograph use decreased modestly with each additional patient: aOR 0.994 (95% CI, 0.990–0.998, *P* < .001), with a cumulative ARD of −1.6 pp. Ultrasound showed a comparable effect: aOR 0.994 (95% CI, 0.990–0.998, *P* < .001), ARD −0.7 pp, with the largest between-physician effect (aOR 0.931), suggesting that high-throughput physicians use US substantially less than low-throughput physicians overall.

Magnetic resonance imaging was nonsignificant in the pooled model (aOR 0.993, 95% CI, 0.984–1.001, *P* = .09) but the GLMM, which properly accounts for physician clustering, detected a significant association (aOR 0.992, *P* < .001; ICC 0.034). The ARD was negligible (−0.1 pp) given the very low base rate (1.0%). This is intuitive since MRI is particularly time-intensive; when busy, physicians may defer MRI to inpatient settings.

Notably, noncontrast CT was the only modality unaffected by workload (aOR 1.000, 95% CI, 0.997–1.004, *P* = .87). This implies that CT commonly ordered for critical diagnoses, such as noncontrast head CT for stroke or trauma, remained steady regardless of physician load. The difference between contrast and noncontrast CT behavior suggests physicians may consciously or unconsciously differentiate: avoiding the more time-consuming CTs (requiring IV access, lab checks, contrast administration) when busy, while still obtaining necessary noncontrast studies.

### Within-Between Decomposition and Mixed-Effects Models

The Mundlak model confirmed that within-physician effects were consistent with pooled estimates for all modalities, supporting the inference that individual physicians change their imaging behavior as workload increases within a shift. Between-physician effects were consistently larger in magnitude (eg, aOR 0.933 versus 0.991 for CT with IV contrast), but Hausman tests were nonsignificant for all modalities (all *P* > .05), meaning we cannot definitively conclude the two mechanisms differ in magnitude. The GLMM random intercept standard deviations ranged from 0.256 (US) to 0.402 (CT with IV contrast), and ICCs ranged from 0.020 to 0.047, indicating that 2–5% of imaging variance is attributable to physician-level differences (Figures 3 and 4).

## DISCUSSION

### Summary of Findings

In this six-year retrospective study of nearly 288,000 ED visits, increasing physician patient load was associated with modest but statistically significant decreases in the proportion of patients receiving imaging, particularly for resource-intensive modalities. CT with IV contrast showed the strongest workload effect (ARD −2.2 pp over a full shift), followed by any CT (−1.9 pp), radiograph (−1.6 pp), and US (−0.7 pp). Noncontrast CT, often ordered for critical diagnoses such as stroke or trauma, was unaffected, suggesting physicians preserve essential imaging even under high workload. While the per-patient effect was small, the cumulative impact over a typical shift was clinically meaningful.

### Interpretation

The modality-specific pattern supports an adaptive rather than indiscriminate response to workload pressure. Computed tomography with contrast and US require additional steps—IV access, creatinine checks, contrast administration time, or technician availability—that may be deferred when time is limited. For example, a physician managing multiple acute patients may choose to observe, arrange outpatient imaging, or admit a patient rather than order an immediate CT abdomen with contrast. Noncontrast CT and radiography are faster to obtain and often protocolized for acute presentations, explaining their relative stability.

The selective nature of this pattern is reassuring from a patient safety perspective: many noncontrast CTs are performed for critical, cannot-miss diagnoses (head CT for trauma or stroke, cervical spine CT for trauma). The fact that physicians did not decrease ordering of noncontrast CTs under high load suggests continued adherence to imaging for high-risk situations even when busy. Physicians appear to be cutting “optional” or less urgent imaging first while preserving necessary emergent imaging.

The within-between decomposition confirmed that these effects operate within individual physicians as workload varies within a shift, though between-physician practice style differences also contribute. Between-physician effects were consistently larger in magnitude (eg, aOR 0.933 vs 0.991 for CT with contrast), suggesting that high-throughput physicians inherently order fewer imaging studies, but Hausman tests were nonsignificant. The ICCs of 0.020–0.047 indicate that 2–5% of imaging variance is attributable to physician-level heterogeneity, justifying mixed-effects modeling for this data structure.

These findings also touch on the efficiency-versus-thoroughness trade-off in emergency medicine. Under high workload, clinicians tend to satisfice, doing just enough to manage the problem, possibly at the expense of thoroughness, eg CT without contrast, which lowers length of stay.^9^ Our data show that as workload increases, thoroughness (in terms of extensive imaging) diminishes slightly, presumably in favor of efficiency.

### Comparison to Previous Studies

To our knowledge, this is among the first studies to directly link physician-specific real-time workload to imaging use in the ED. Prior investigations into ED crowding have largely focused on system-level outcomes rather than individual physician practice patterns. Our findings align with evidence that more experienced, higher-throughput physicians use slightly less CT imaging.^9^ The efficiency-thoroughness trade-off, a well-recognized concept in human factors engineering, is consistent with our observation that diagnostic thoroughness (imaging) diminishes slightly as workload increases, presumably in favor of efficiency.

Notably, the direction of our finding runs opposite to most published work on emergency physician workload and imaging use. Prior studies, many conducted by radiology investigators using department-wide volume metrics, have generally reported that imaging orders increase when emergency physicians are busier or when the ED itself is more crowded.^10^–^12^ The dominant explanation in that literature has been a substitution hypothesis: Under time pressure, physicians lean on imaging as a faster proxy for a comprehensive history and physical examination, “ordering tests to think.”

Several important methodological differences likely account for our opposite finding. First, the prior literature largely measured department-level crowding (ED occupancy, boarding burden, total daily volume) rather than the physician-specific real-time patient load we use here, and department-level metrics can confound patient mix and ordering behavior in ways that bias toward more imaging. Second, much of that work was performed before contemporary throughput-focused operational pressures became routine in academic EDs; physician behavior under crowding has plausibly changed as length-of-stay metrics, value-based care incentives, and Choosing Wisely campaigns have matured. Third, our outcome is imaging use per individual patient encounter, normalized against case mix, rather than absolute imaging volume; volume-based metrics conflate higher patient throughput with higher imaging because both naturally rise together.

Read in this light, our results suggest that, at our institution, imaging is not functioning as a substitute for a careful history and physical examination when physicians are under workload pressure. If imaging were being used as a cognitive shortcut, we would expect to see the prior literature’s pattern of increased ordering with higher workload, especially for the resource-intensive modalities that take time to perform. We see the opposite. The modalities that are most resource-intensive to obtain (CT with IV contrast, US) showed the largest workload-associated decreases, while the modalities that are typically protocolized and fast to obtain (noncontrast CT, plain radiography) showed the smallest or no decreases. This pattern is more consistent with physicians selectively deferring optional or slower-to-resource imaging when they have less time per patient, while preserving cannot-miss imaging for high-risk presentations. Practically, this implies that increasing emergency physician workload is not driving overuse of imaging in our setting; rather, it is associated with selective de-use that is concentrated in modalities where delay is least likely to cause immediate harm. Whether this selective de-use is clinically appropriate (omitting marginal-value imaging that does not change management) or clinically concerning (omitting imaging that would have altered care) cannot be determined from this study and is the most important question for future prospective work.

### Strengths

Key strengths include a large sample size (287,925 encounters), objective workload measurement via automated rotational patient assignment (which removes selection bias in patient allocation), six years of longitudinal data, three complementary analytic approaches (pooled logistic, GLMM, and Mundlak decomposition), and adjustment for chief complaint alongside standard confounders including triage acuity and temporal factors.

### Clinical Implications

Clinicians should be aware that workload pressure may subtly alter imaging thresholds. During peak volume periods, decision-support tools or imaging safety nets (eg, automated prompts for high-risk presentations) may help ensure necessary studies are not omitted. Conversely, the finding that imaging decreases under pressure, without evidence of harm, raises the possibility that some imaging ordered during low-workload periods may be of marginal value, aligning with broader efforts to reduce imaging overuse in emergency medicine.

From an operational standpoint, our results suggest that imaging demand per patient is not static but can drop during peak workload conditions. This could temporarily alleviate pressure on radiology but may also mean deferred studies increase demand on inpatient wards. Department leaders should note that providing safety nets during crowding is crucial to ensure quality of care is maintained under variable workload conditions.

### Sign-Out Culture and End-of-Shift Behavior

An additional factor that may contribute to reduced imaging later in a physician’s shift is the culture surrounding clinical sign-outs. At our institution, as at many EDs, physicians at the end of their shifts transfer care of remaining patients to the incoming physician. If ordering a resource-intensive imaging study, such as CT with IV contrast, near the end of a shift means the ordering physician will not be present to interpret and act on the results, they may be less inclined to initiate such studies. This reluctance could stem from a desire to avoid burdening the incoming physician with pending results, or from concerns about continuity of care when the clinical context is lost during handoff. Conversely, at institutions where sign-out of pending studies is routine and well-integrated into workflow, this effect may be attenuated. Our study design did not allow us to distinguish between workload-driven behavioral adaptation and end-of-shift sign-out avoidance, as both factors increase with higher assignment order. Future studies incorporating shift-end timing data could help disentangle these mechanisms.

### Research Implications

Future research should link workload-related imaging changes to patient outcomes (eg, missed diagnoses, return ED visits, delayed treatment) and extend to multicenter settings to assess generalizability across institutions with differing patient assignment systems. Whether computerized decision support for imaging (eg, clinical decision rules built into order entry) reduces workload-related variation warrants investigation. Additionally, examining whether other clinical behaviors (lab test ordering, consultations) follow a similar workload pattern would broaden understanding of how crowding affects emergency care.

## LIMITATIONS

This was a single-center observational study at an academic tertiary care center, which may limit generalizability. The rotational patient assignment system is not universal; institutions where physicians have discretion in self-assigning patients may show different patterns due to potential selection bias. Physician assignment order is an imperfect proxy for simultaneous patient load and does not capture other workload aspects such as patient acuity load or interruptions. As discussed in the section “Sign-Out Culture and End-of-Shift Behavior,” because higher assignment order is correlated with proximity to end of shift, we cannot fully disentangle workload-driven behavioral adaptation from end-of-shift sign-out avoidance; both factors may contribute to the modality-specific patterns observed.

We lacked granular clinical data (eg, comorbidities, secondary chief complaints) that influence imaging decisions, particularly at a center with an elevated median age and a high percentage of immunocompromised patients. We included triage acuity and chief complaint to help mitigate this. The study period (January 2019–December 2024) overlapped substantially with the COVID-19 pandemic, which altered both ED volume and imaging mix at our institution; a prior analysis from our group documented sharp shifts in contrast-CT use driven by intermittent contrast shortages during this period. While our primary analysis adjusts for temporal factors and the consistent modality-specific pattern argues against pandemic-driven artifact as the dominant explanation, residual pandemic-era confounding cannot be excluded.

Imaging-resource availability also varies systematically by shift (radiology-technician staffing, MRI availability, consultant access), and we were unable to measure or adjust for these system-level factors directly. Because our outcome is imaging ordered rather than imaging clinically indicated, we have no direct measure of the appropriateness of each study, and we did not assess patient outcomes (return visits, missed diagnoses, mortality); whether reduced imaging under high workload represents safe efficiency or poses diagnostic risk therefore remains unknown and is the most important question for future prospective work. Point-of-care US performed without formal orders may be undercaptured, although this likely does not systematically differ by workload. We did not adjust for multiple comparisons across six outcomes; however, the consistent pattern (five of six modalities in the same direction) supports genuine associations rather than spurious findings.

## CONCLUSION

Higher physician workload was associated with modest reductions in imaging use, particularly for resource-intensive modalities such as CT with IV contrast and ultrasound. Noncontrast CT was unaffected, suggesting physicians preserve essential imaging even when busy. A within-between decomposition supported the interpretation that these effects reflect real-time behavioral adaptation within individual physicians. These findings highlight a trade-off between ED throughput and diagnostic testing that warrants further study regarding its impact on clinical outcomes.

## Supplementary Information



## Figures and Tables

**Figure 1:**
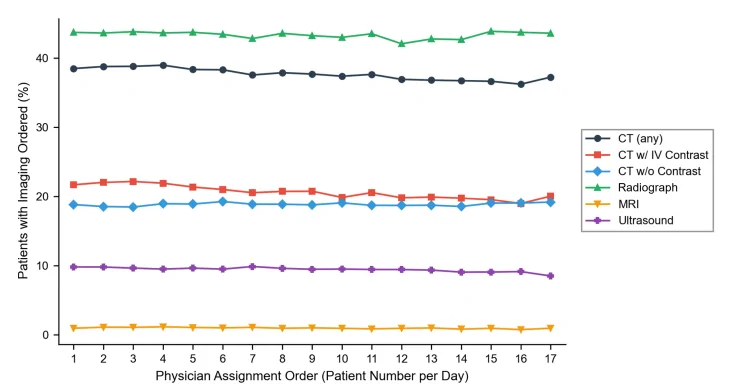
Unadjusted imaging use by order of physician patient assignment, in a study of 287,925 emergency department encounters examining the association between physician workload and diagnostic imaging ordering (January 2019–December 2024). *CT*, computed tomography; *IV*, intravenous; *MRI*, magnetic resonance imaging.

**Figure 2:**
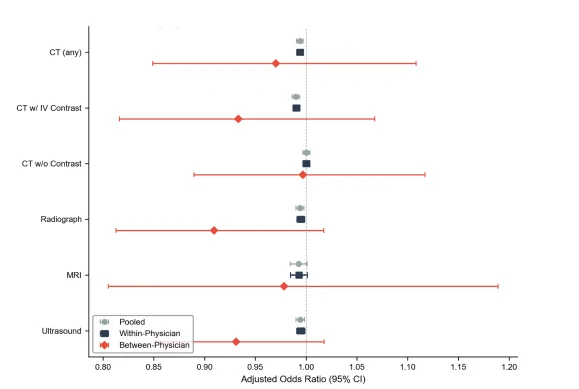
Pooled vs within-physician vs between-physician effects on imaging use per unit increase in workload, in a study of 287,925 emergency department encounters evaluating physician workload and imaging use. *CT*, computed tomography; *IV*, intravenous; *MRI*, magnetic resonance imaging.

**Figure 3:**
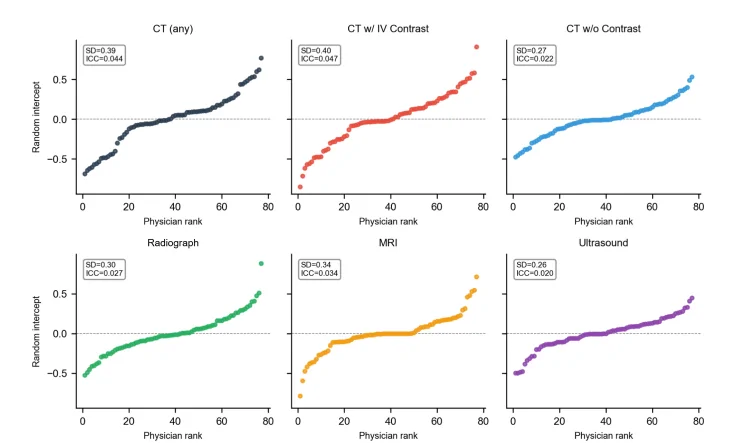
Physician random intercepts by imaging modality, in a study of 287,925 emergency department encounters across 77 physicians examining workload effects on imaging ordering. *CT*, computed tomography; *IV*, intravenous; *MRI*, magnetic resonance imaging.

**Figure 4:**
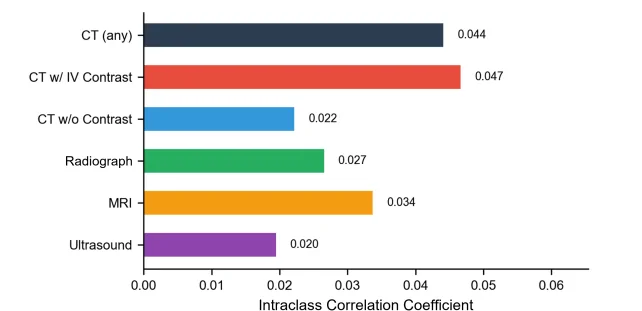
Physician-level intraclass correlation coefficient for each imaging modality, in a study of 287,925 emergency department encounters evaluating between-physician variance in imaging use. CT, computed tomography; IV, intravenous; MRI, magnetic resonance imaging.

**Table 1:** Adjusted odds of imaging use per one-patient increase in physician daily workload, in a study of 287,925 emergency department encounters at an academic tertiary care center (January 2019–December 2024).

Imaging Modality	Pooled aOR (95% CI)	Pooled *P* value	Within-Physician aOR	Between-Physician aOR	ICC	ARD (pp)
CT (any)	0.994 (0.991–0.997)	< .001	0.994	0.940	0.044	−1.9
CT with IV contrast	0.990 (0.986–0.994)	< .001	0.991	0.933	0.047	−2.2
CT without contrast	1.000 (0.997–1.004)	.87	1.000	0.960	0.022	0.0
Plain radiography	0.994 (0.990–0.998)	.003	0.994	0.966	0.027	−1.6
MRI	0.993 (0.984–1.001)	.087	0.992*	0.955	0.034	−0.1
US	0.994 (0.990–0.998)	.006	0.994	0.931	0.020	−0.7

* Significant in generalized linear mixed model (*P* < .001) but not pooled model.

*aOR*, adjusted odds ratio; *ARD*, absolute risk difference (pp = percentage points, 17th vs first patient); *CT*, computed tomography; *ICC*, intraclass correlation coefficient; *MRI*, magnetic resonance imaging; *US*, ultrasound.
