# Supplementary material for: Impact of Physician Patient Load on Imaging Use in the Emergency Department
Source: West J Emerg Med. 2026 Sep 24;27(5):1208–16. doi: 10.5811/westjem.63096 (PMC13660594; doi:10.5811/westjem.63096)
Supplement: Supplementary file 1 [file wjem-27-1208-S1.pdf]

SUPPLEMENTARY MATERIAL

Impact of Physician Patient Load on Imaging Use in the Emergency Department

Martini WA, Monas J, Hodgson NR. West J Emerg Med. 2026;27(5). DOI 10.5811/westjem.63096

Supplementary Table S1. Adjusted odds ratios for all covariates in the six pooled multivariable logistic regression models of imaging use, in a study of 287,925 emergency department encounters at an academic tertiary care center (January 2019–December 2024).

Covariate	CT (any) aOR (95% CI)	CT with IV contrast aOR (95% CI)	CT without contrast aOR (95% CI)	Plain radiography aOR (95% CI)	MRI aOR (95% CI)	US aOR (95% CI)
Physician assignment order, per additional patient	0.994 (0.991–0.997)	0.990 (0.987–0.993)	1.000 (0.997–1.004)	0.994 (0.990–0.998)	0.992 (0.983–1.000)	0.994 (0.990–0.998)
Patient characteristics						
Age, per 10 years	1.13 (1.11–1.14)	1.05 (1.04–1.06)	1.15 (1.14–1.16)	1.13 (1.12–1.14)	1.03 (1.01–1.05)	0.96 (0.95–0.96)
Male sex (vs female)	0.94 (0.92–0.97)	0.94 (0.92–0.96)	0.98 (0.96–1.01)	1.05 (1.03–1.07)	1.09 (1.01–1.16)	0.89 (0.86–0.92)
Other or unknown sex (vs female)	1.16 (0.46–2.97)	1.51 (0.54–4.25)	0.99 (0.26–3.81)	0.84 (0.32–2.25)	NE	0.81 (0.23–2.87)
Triage acuity						
ESI 1 (vs ESI 3)	3.81 (3.32–4.38)	3.38 (2.95–3.86)	2.29 (2.06–2.55)	1.40 (1.26–1.56)	10.12 (8.65–11.84)	0.25 (0.19–0.32)
ESI 2 (vs ESI 3)	1.39 (1.34–1.44)	1.20 (1.15–1.25)	1.49 (1.44–1.54)	1.59 (1.51–1.67)	1.78 (1.64–1.94)	0.94 (0.89–0.99)
ESI 4 (vs ESI 3)	0.26 (0.25–0.27)	0.19 (0.18–0.20)	0.39 (0.36–0.42)	1.27 (1.21–1.35)	0.44 (0.38–0.53)	0.62 (0.59–0.65)
ESI 5 (vs ESI 3)	0.02 (0.01–0.04)	0.02 (0.01–0.04)	0.03 (0.01–0.06)	0.12 (0.10–0.15)	0.14 (0.04–0.50)	0.05 (0.03–0.08)
ESI not recorded (vs ESI 3)	0.23 (0.14–0.37)	0.32 (0.17–0.59)	0.28 (0.16–0.49)	0.36 (0.23–0.54)	NE	0.28 (0.14–0.57)
Chief complaint (top 10)						
Abdominal pain (vs other complaint)	5.00 (4.63–5.40)	6.50 (6.05–6.98)	0.90 (0.83–0.98)	0.39 (0.36–0.43)	0.24 (0.19–0.31)	1.69 (1.60–1.79)
Chest pain (vs other)	0.72 (0.67–0.78)	1.61 (1.48–1.74)	0.20 (0.19–0.22)	13.47 (11.37–15.96)	0.17 (0.13–0.21)	0.42 (0.38–0.45)
Shortness of breath (vs other)	0.90 (0.84–0.96)	1.93 (1.79–2.07)	0.28 (0.25–0.31)	10.82 (9.28–12.61)	0.53 (0.40–0.69)	0.83 (0.78–0.89)
Fall (vs other)	4.46 (3.95–5.03)	0.44 (0.38–0.51)	7.83 (6.92–8.87)	3.03 (2.80–3.27)	1.03 (0.84–1.27)	0.15 (0.13–0.17)
Fever (vs other)	0.79 (0.74–0.84)	1.18 (1.12–1.26)	0.52 (0.47–0.58)	5.99 (5.40–6.66)	0.86 (0.69–1.06)	0.64 (0.57–0.71)
Back pain (vs other)	2.22 (1.93–2.55)	1.13 (1.05–1.22)	2.75 (2.36–3.21)	0.78 (0.68–0.88)	7.35 (6.28–8.60)	0.36 (0.33–0.40)
Headache (vs other)	3.34 (3.01–3.71)	1.30 (1.12–1.51)	3.92 (3.58–4.30)	0.50 (0.47–0.54)	1.98 (1.59–2.47)	0.12 (0.09–0.15)
Cough (vs other)	0.48 (0.44–0.52)	0.84 (0.75–0.93)	0.30 (0.25–0.35)	11.55 (9.93–13.43)	0.47 (0.31–0.71)	0.17 (0.15–0.20)
Dizziness (vs other)	1.64 (1.51–1.79)	1.57 (1.38–1.79)	1.38 (1.24–1.53)	1.14 (1.00–1.30)	1.83 (1.46–2.30)	0.24 (0.20–0.28)
Generalized weakness (vs other)	1.19 (1.11–1.26)	0.90 (0.85–0.95)	1.38 (1.28–1.49)	3.23 (2.90–3.58)	1.90 (1.58–2.28)	0.58 (0.52–0.64)
Temporal factors						
Arrival hour, per hour (0–23)	1.00 (0.99–1.00)	1.00 (0.99–1.00)	1.00 (1.00–1.01)	1.00 (1.00–1.01)	0.98 (0.97–0.99)	1.00 (1.00–1.01)
Night-shift arrival (≥ 10:30 pm)	1.02 (0.93–1.12)	1.02 (0.89–1.17)	1.04 (0.92–1.18)	0.97 (0.87–1.08)	0.72 (0.55–0.94)	0.91 (0.82–1.02)
Weekend arrival	0.96 (0.93–1.00)	0.96 (0.92–1.00)	0.98 (0.95–1.01)	1.01 (0.98–1.05)	0.83 (0.74–0.92)	0.95 (0.92–0.98)
Model summary						
Encounters in model, n	287,925	287,925	287,925	287,925	287,925	287,925
Imaging rate, %	37.9	20.9	18.8	43.4	1.0	9.5
c-statistic	0.710	0.720	0.717	0.766	0.740	0.653

Six separate pooled logistic regression models were fit, one per imaging modality, each including all covariates shown, with Huber-White robust standard errors clustered by attending physician; 95% CIs that exclude 1.00 correspond to $P < .05$ (two-tailed). The first row is the workload effect reported in Table 1. Age was modeled as a continuous variable and is expressed per 10-year increase. Reference categories: female sex; ESI 3; chief complaint other than the ten most frequent presenting complaints; weekday arrival. Sex recorded as nonbinary or unknown (n = 21) and ESI not recorded (n = 182) are retained as indicator variables; for MRI these two indicators could not be estimated because no encounter in either category received MRI (NE), and they were omitted from that model. The c-statistic is the area under the receiver operating characteristic curve for each model. Models were re-estimated for this supplement from the analytic data set; workload estimates agree with Table 1 to within ±0.001. aOR, adjusted odds ratio; CI, confidence interval; CT, computed tomography; ESI, Emergency Severity Index; IV, intravenous; MRI, magnetic resonance imaging; NE, not estimable; US, ultrasound.

Supplementary Figure S1. Unadjusted imaging use by exact physician assignment order (1–17) with 95% confidence bands, in a study of 287,925 emergency department encounters examining the association between physician workload and diagnostic imaging ordering (January 2019–December 2024).

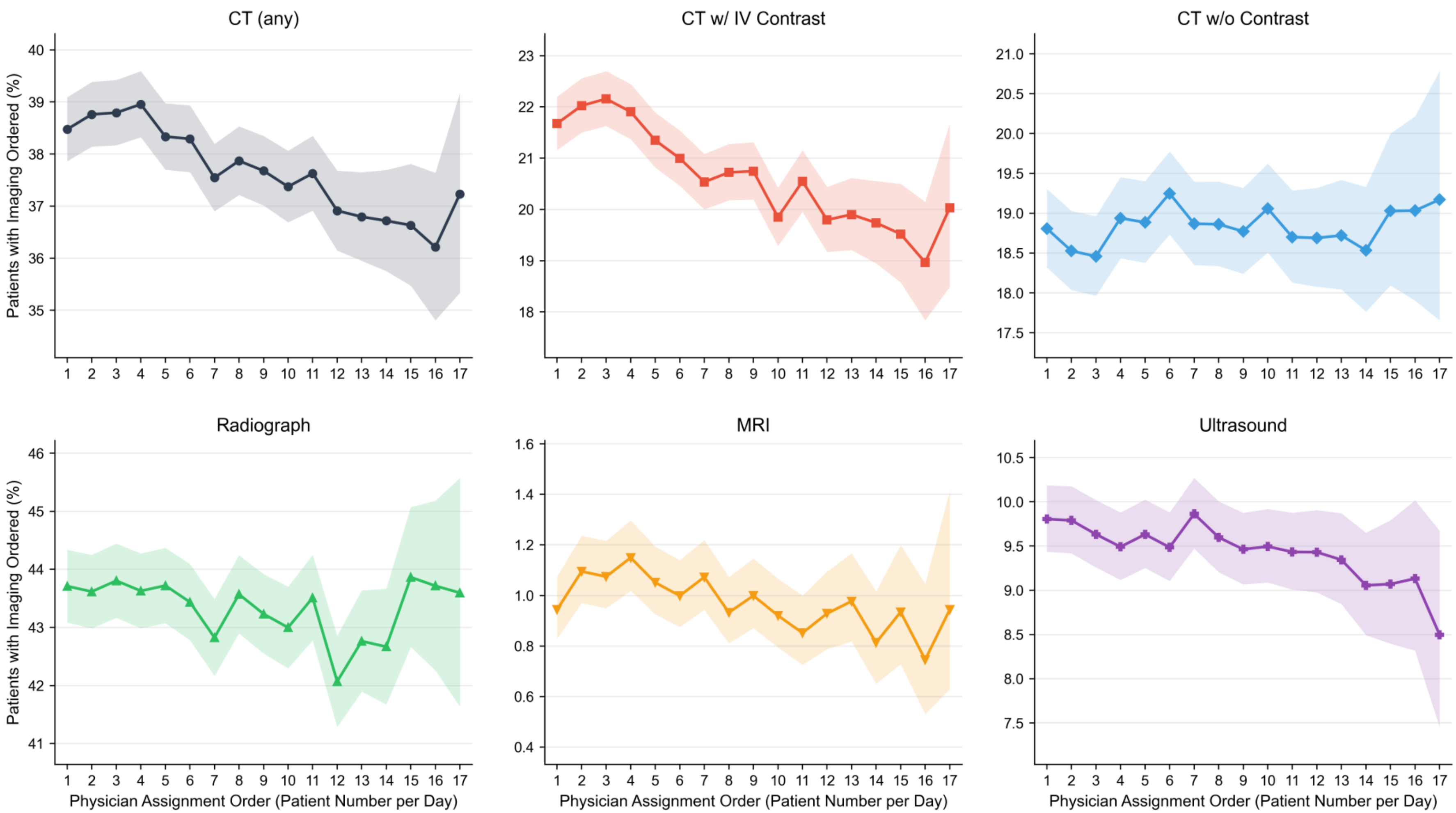

Each panel shows, for one imaging modality, the proportion of encounters in which that modality was ordered at each physician assignment order from 1 to 17 (markers and line), with the shaded band giving the 95% Wilson score confidence interval for each proportion. Panels complement Figure 1, which plots the same data by assignment-order grouping. Encounters per assignment order ranged from 24,161 (order 1) to 2,436 (order 17). CT, computed tomography; IV, intravenous; MRI, magnetic resonance imaging.
